# Type II Kounis syndrome triggered by iodinated contrast during coronary angioplasty: A case report and clinical insights

**DOI:** 10.1016/j.ijscr.2025.111614

**Published:** 2025-07-05

**Authors:** Mohamad Darwish, Rima Chaddad, Syed Muhammad Hassan, Allahdad Khan, Mohamad El Kasty, Jamil Nasrallah

**Affiliations:** a Department of Cardiology, Grand Hôpital de l'Est Francilien, France; b Karachi Medical and Dental College, Karachi, Pakistan; c Nishtar Medical University, Multan, Pakistan; d Department of Medicine, Faculty of Medical Sciences, Lebanese University, Beirut, Lebanon; e Department of Life Sciences, Faculty of Sciences, Lebanese University, Beirut, Lebanon

**Keywords:** Kounis syndrome, Type II, Anaphylaxis, Tryptase, Hypersensitivity, Coronary spasm, Angioplasty

## Abstract

**Introduction and importance:**

Kounis syndrome or “allergic angina” or “allergic myocardial infarction” is acute coronary syndrome that results from hypersensitivity reactions. It may also result from interventions involving the use of iodinated contrast media in patients with previous coronary artery disease. Early treatment and diagnosis prevent severe cardiovascular implications.

**Case presentation:**

We present the case of a 57-year-old male patient with a history of ischemic heart disease who developed Type II Kounis syndrome during a scheduled coronary angioplasty. On administration of iodinated contrast (Visipaque), the patient suddenly experienced anaphylactic shock, diffuse coronary vasospasm, ST elevation, and acute left ventricular failure. Although he did not have any known history of previous allergies, the clinical presentation was consistent with acute hypersensitivity reaction superimposed on atherosclerotic disease. Immediate treatment with intravenous adrenaline, corticosteroids, antihistamines, and hemodynamic stabilization resulted in rapid clinical stabilization.

**Clinical discussion:**

Coronary angiography was found to reveal complete reversal of coronary spasm after treatment. Follow-up laboratory tests demonstrated a stark rise in the concentration of serum tryptase during the acute attack, followed by normalization, which confirmed mast cell activation. The patient was discharged in stable condition with cardiology and allergology follow-up, including contrast allergy test.

**Conclusion:**

Kounis syndrome is an uncommon but life-threatening condition to be remembered in patients with pre-existing coronary artery disease undergoing contrast-related procedures and developing features of anaphylaxis. This case report highlights the clinical presentation of Type II Kounis syndrome, and awareness and multidisciplinary approach need to be increased for optimal outcome.

## Highlights

• A rare but serious condition where an allergic reaction coexists with acute coronary syndrome, leading to coronary vasospasm triggered by inflammatory mediators.
• A 57-year-old male with ischemic heart disease developed acute symptoms, including hypotension, skin rash, and coronary vasospasm shortly after iodine contrast injection during coronary angiography.
• The patient was diagnosed with Type II Kounis Syndrome, where allergic-mediated coronary vasospasm occurred on top of pre-existing atherosclerotic coronary disease.
• The patient received immediate treatment with adrenaline, corticosteroids, antihistamines, and furosemide. Coronary spasm resolved after treatment, and the patient showed significant improvement.
• Tryptase levels decreased from 17 μg/L to 3.2 μg/L, confirming mast cell deactivation. Further iodine contrast allergy tests were planned to confirm the cause of the allergic reaction.

## 1 Introduction

Kounis syndrome (KS) is a rare but potentially life-threatening condition involving the co-existence of an allergic reaction and acute coronary syndrome to an allergenic event [1]. It represents an overlap of cardiovascular and immunologic responses where inflammatory mediators generated during an allergic reaction cause coronary vasospasm. Approximately 1.1 % of patients hospitalized for allergic, hypersensitivity or anaphylactic reactions are diagnosed with KS in the United States [2]. This corresponds to approximately 19.4 cases per 100,000 emergency department visits [3]. KS can present in any age group, though it is most prevalent in adults aged 40 to 70, covering 68 % of cases [3]. It occurs more frequently in males, who constitute 74.3 % of cases, as opposed to females at 25.7 % [3]. This is a serious condition with high risk, from cardiac arrest in 6.3 % of the cases and death in 2.9 % of the cases [3].

Though Kounis syndrome has been described worldwide, it is most frequently described in the southern European nations, especially Turkey, Greece, Italy, and Spain [4]. The incidence that has been reported may be lower than its actual incidence since it may still be underdiagnosed or misdiagnosed. Therefore, proper awareness and diagnosis are needed in managing this potentially lethal illness [1,3]. KS is of three types based on its pathophysiology. Type I occurs in patients without previous coronary artery disease and is the result of vasospasm secondary to allergic mediators. Type II occurs in the setting of previous atherosclerotic disease in which there is an allergic reaction leading to plaque rupture and myocardial infarction. Type III involves stent thrombosis secondary to hypersensitivity reactions [5]. Mast cell activation, histamine release, and inflammatory mediators play key roles in KS. These common triggers are drugs, foods, and insect bites [5].

This case reports a 57-year-old man with pertinent medical history. Apart from ischemic heart disease, inguinal hernia, gout, smoking, early family history of CAD, and Overweight, not previously known to have any previous allergy and came for a planned angioplasty. It discusses his clinical presentation, diagnostic workup, management, and follow-up strategy, highlighting the identification and early management of type II KS.

This manuscript was prepared following the SCARE guidelines [6].

## 2 Case presentation

### 2.1 Patient background

A 57-year-old male patient with a history of ischemic heart disease presented to the hospital for an elective coronary angiogram. His history included anterior myocardial infarction, which had been treated with stenting of the left anterior descending (LAD) artery and an implantable cardioverter-defibrillator (ICD) for secondary prevention. His cardiovascular risk factors were a family history of premature CAD, smoking 5–6 cigarettes/day, and obesity (BMI = 30.8). On admission, a routine electrocardiogram (ECG) was performed, which revealed a sinus rhythm with left axis deviation, Q waves in aVL, and negative T waves in aVL and lead I, consistent with the patient's history of anterior myocardial infarction.

### 2.2 Procedure and immediate reaction

**Fig. 1:**
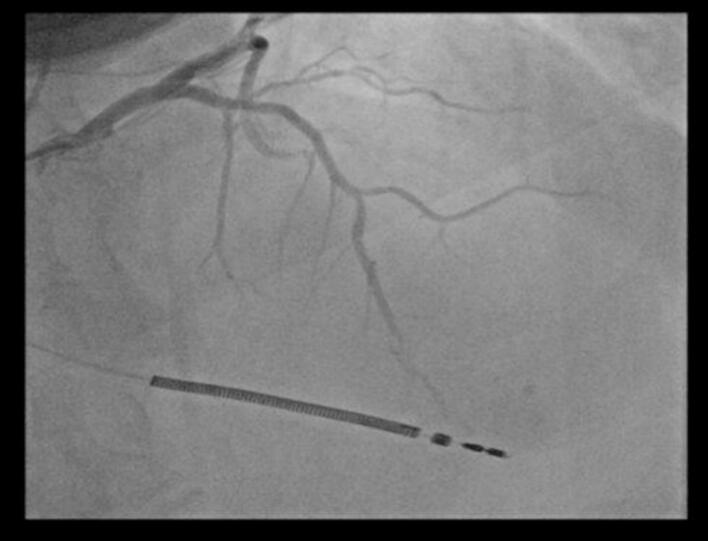
Coronary angiography showing significant stenosis of the distal LAD with a very pathological downstream bed.

**Fig. 2:**
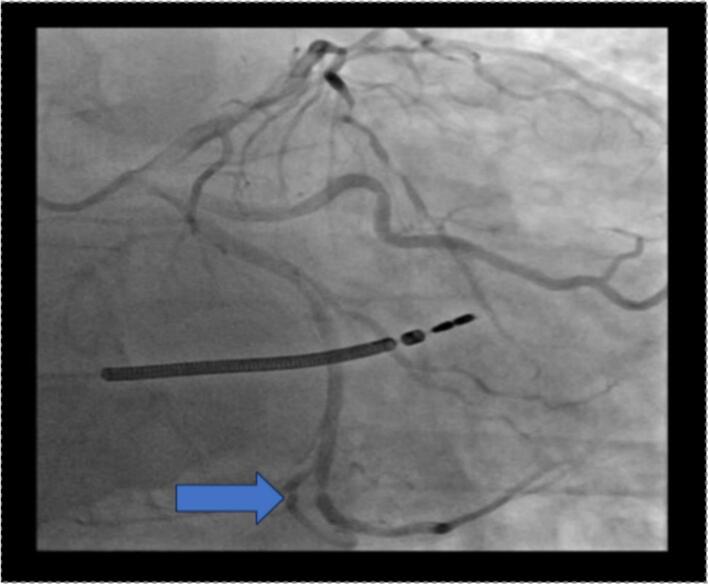
Coronary angiography showing significant stenosis of the distal circumflex artery.

**Fig. 3:**
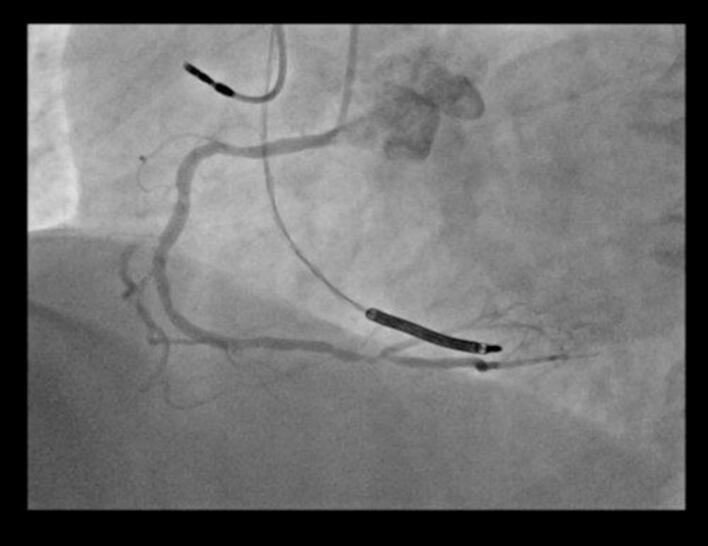
Coronary angiography significant stenosis of the distal right coronary artery.

Coronary angiography using Visipaque (iodine-based contrast) demonstrated a patent LAD stent with no signs of restenosis. However, severe stenosis was observed in the distal LAD (which was of diffusely narrowed caliber with a poor distal vessel bed), the distal circumflex artery, and the distal right coronary artery (Fig. 1, Fig. 2, Fig. 3). Based on a Syntax Score of 21, indicating intermediate coronary complexity, PCI was performed selectively on the distal circumflex artery using a drug-eluting stent, which presented the most hemodynamically significant lesion amenable to stenting.

**Fig. 4:**
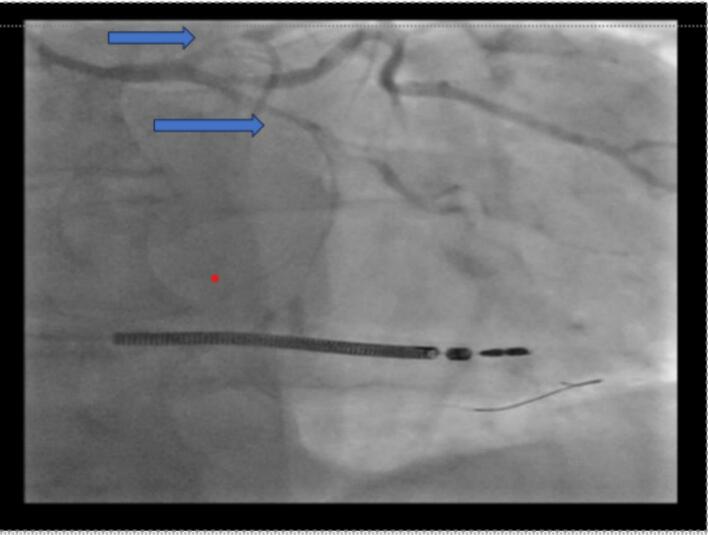
Coronary angiography showing diffuse coronary spasm (CX and LAD).

Shortly after contrast administration, the patient developed acute refractory hypotension, generalized erythematous rash, global ST-segment elevation, and signs of acute left ventricular failure. Coronary angiography at that moment revealed diffuse coronary vasospasm (Fig. 4), raising concern for Kounis syndrome triggered by contrast hypersensitivity.

### 2.3 Management

**Fig. 5:**
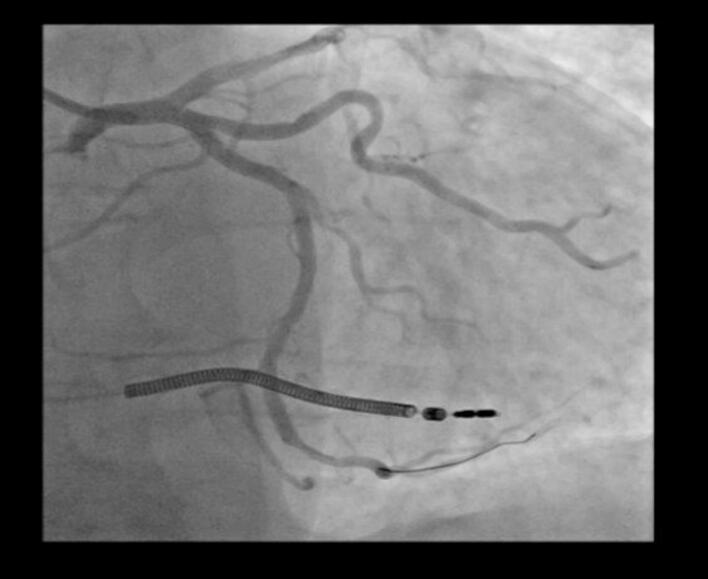
Showing final of coronary angioplasty after treatment of coronary spasm.

Immediate resuscitative measures included intravenous administration of an adrenaline bolus dose of 0.1 mg and intravenous corticosteroids in the form of Solupred 120 mg. Given the presence of hypotension, diffuse coronary vasospasm, generalized rash alongside ST elevation, the differential diagnosis considered cardiogenic shock due to allergic vasospasm of the coronary arteries. The rapid onset after contrast suggested anaphylaxis. Hypotensive, the patient was still given intravenous furosemide (Lasix) and dobutamine because of pulmonary edema as well as acute decompensated heart failure. An adrenaline infusion, initiated at 0.4 mg/h, was maintained. Blood pressure improved from 75/40 mmHg to 105/65 mmHg within 20 min of initiating the infusion. Corticosteroids and antihistamines were administered to inhibit the hypersensitivity reaction. A follow-up coronary angiogram revealed resolution of the coronary spasm after treatment (Fig. 5).

### 2.4 In-hospital course

Given the patient's history of coronary artery disease and coronary vasospasm secondary to an allergic stimulus, the presentation was consistent with Type II Kounis syndrome, which is allergic-mediated coronary vasospasm superimposed over existing atherosclerotic disease. The patient was admitted to the coronary care unit (CCU) for observation, where echocardiography revealed a left ventricular ejection fraction (LVEF) of 35 %, antero-septo-apical akinesia, and left ventricular dilation (LVDD 65 mm), all consistent with acute ischemic myocardial dysfunction superimposed on chronic ischemic heart disease.

### 2.5 Follow-up and discharge

Management of both allergic and cardiac dimensions of Kounis syndrome and included beta-blockers, adrenaline, corticosteroids, and antihistamines. The patient presented with significant improvement after treatment and all medications, especially adrenaline, were tapered out gradually. With high suspicion for contrast-induced hypersensitivity, consultation with an allergologist was organized, and the patient was discharged with a pre-planned schedule for follow-up. Laboratory tests after discharge indicated a profound reduction of the tryptase level, from 17 μg/L at the time of reaction to 3.2 μg/L, confirming mast cell deactivation. Latex-specific IgE was also negative (<0.1 kU/L) and so ruled out latex as a potential allergen. Further confirmation tests for iodine contrast allergy were recommended six weeks post-discharge, including planned skin prick and intradermal testing under allergologist supervision.

## 3 Discussion

Kounis syndrome is the name given to the overlap between acute coronary syndromes and hypersensitivity reactions, first described in 1991. It is currently classified into three types: Type I is coronary spasm without proven coronary disease, Type II is in patients with established atherosclerosis in whom allergic reactions lead to rupture of plaque or coronary vasospasm, and Type III is with in-stent thrombosis due to hypersensitivity [7]. This case offers significant clinical insights regarding how mast cell activation during an allergic reaction can lead to coronary vasospasm in patients with atherosclerosis. The real-time angiographic demonstration of diffuse coronary spasm and its later resolution after treatment is especially telling. The pathophysiology behind Type II Kounis syndrome is activation and degranulation of mast cells with discharge of various vasoactive and pro-thrombotic mediators such as histamine, tryptase, chymase, leukotrienes, and platelet-activating factor [8]. These have a direct effect on coronary vasculature to cause vasospasm and additionally break down the fibrous cap of atheromatous plaques, leading to destabilization and potential rupture of the plaque [8]. The pro-thrombotic and pro-inflammatory state induced during anaphylaxis favors intravascular thrombosis, causing myocardial infarction or ischemia.

Clinically, the diagnosis is often overlooked due to overlap with classic ACS presentations, particularly in already-high-risk patients for coronary artery disease. However, the presence of allergic manifestations like urticaria, hypotension, bronchospasm, or flushing accompanied by ischemic chest pain should clinically raise suspicion [8]. In this case, the diagnostic process involved correlating sudden systemic allergic symptoms with ECG changes and angiographic findings. The serum level of tryptase confirmed mast cell activation and, thus, an allergic cause. The management of epinephrine, steroids, and antihistamines given in steps was based on the latest protocols for anaphylactic reactions with heart involvement. In our patient, hypotension, generalized rash, and acute onset of diffuse ST-segment elevation a few seconds following contrast exposure were key diagnostic hints. The duration of serum tryptase at the time of the acute event provided biochemical proof of mast cell degranulation. According to consensus criteria, acute mast cell activation is confirmed when serum tryptase increases by ≥(1.2 × baseline + 2 μg/L) within 1–4 h, which in our patient was exceeded [9]. In our case, a level of 17 μg/L during the reaction, followed by 3.2 μg/L in convalescence, confirmed this. Coronary angiography revealed diffuse coronary spasm in the absence of stent restenosis or thrombus, establishing the diagnosis of Type II KS [8].

Type II Kounis syndrome is treated therapeutically by addressing the allergic reaction and myocardial ischemia in a balanced. In this case, we chose to treat both simultaneously with intravenous low-dose adrenaline, corticosteroids, antihistamines, vasopressors, and loop diuretics to stabilize the patient. The offending lesion in the circumflex artery was managed with immediate PCI. However, treatment must be individually tailored with care: beta-blockers are contraindicated, especially non-selective beta-blockers, as they may inhibit the β2-mediated bronchodilation and reduce responsiveness to epinephrine, potentially exacerbating hypotension and bronchospasm. In our case, beta-blockers were withheld during the acute phase and reintroduced later under close monitoring, while morphine is contraindicated since it possesses mast cell–activating activity [10]. Nitrates and calcium channel blockers can be employed to relieve coronary spasm but with caution in hypotension [11]. Although aspirin is a pillar of ACS management, its capacity to exacerbate allergic reactions through leukotriene mechanisms is tempered by the need for wise risk-benefit decision-making [10]. Epinephrine remains the first-line drug of choice for anaphylaxis but, in Kounis syndrome, will need to be slowly titrated so as not to increase demand for myocardial oxygen and augment ischemia [11]. Corticosteroids and H1/H2 receptor antagonists can be used to interrupt the allergic cascade and prevent the delayed-phase reaction [11]. Patients with known allergies undergoing PCI are at increased risk of developing Kounis syndrome, particularly when iodinated contrast agents are used. Risk stratification, pre-procedural screening for prior hypersensitivity, and consideration of premedication are vital preventive steps. While epidemiologic data are limited, certain reports suggest under recognition of this condition in high-risk patients.

From an interventional point of view, this case emphasizes the need for heightened vigilance for Kounis syndrome among cardiologists, particularly in the setting of invasive procedures with contrast media. Early recognition of allergic manifestations, rapid distinction from usual ACS, and timely hemodynamic support can significantly influence outcomes. Ready access to epinephrine, steroids, and antihistamines in the Cath lab is essential. In high-risk patients, such as those with prior allergic reactions to contrast, prophylactic premedication should be considered [8]. Allergologic referral after the event is crucial to identify the causative allergen and to develop preventive measures for future procedures. In our patient, follow-up tryptase levels normalized, and allergy workup for contrast sensitivity was scheduled. Type II Kounis syndrome is a so far underestimated but clinically significant entity in interventional cardiology. This case highlights the necessity for a multidisciplinary strategy integrating acute cardiac treatment and allergy management. Early detection and customized treatment are crucial to optimize clinical outcomes and prevent recurrence in susceptible individuals [8]. Preventive strategies, such as premedication with corticosteroids and antihistamines or use of iso-osmolar contrast agents, should be considered for future interventions. Documentation of contrast allergy and a medical alert tag may be warranted. For patients presenting outside of catheterization labs, the diagnosis of Kounis syndrome should rely on recognition of concurrent hypersensitivity symptoms and cardiac ischemia, supported by biomarkers like serum tryptase, ECG changes, and non-invasive imaging when possible. Our case adds to a limited but growing body of literature documenting contrast-induced Type II Kounis syndrome. In a review by Abdelghany et al., contrast agents were among the most frequently implicated triggers in Type II cases involving atherosclerotic plaque destabilization.

## 4 Conclusion

Kounis syndrome, particularly Type II, represents an important crossroads between acute coronary syndromes and allergic reactions in individuals with the substrate of atherosclerotic disease. The case demonstrates the manner in which elevated suspicion of Kounis syndrome must remain in the clinician's mind during the performance of coronary interventions, especially during hypersensitivity reactions occurring in immediate temporal association with allergenic challenge such as with iodinated contrast. Early prompt identification, immediate implementation of dual-pathway treatment targeting allergic and cardiac aspects alike, and close follow-up after the event are all essential to achieve optimal results. Interventricular cardiologists and emergency physicians should be well aware of this rare but potentially lethal syndrome in order to allow proper diagnosis, individually tailored treatment, and prevention of recurrence through allergologic testing and procedural planning.

**Table:** 

Feature	Type I	Type II	Type III
Coronary status	Normal	Atherosclerotic	Stent-related
Mechanism	Vasospasm	Plaque rupture/spasm	Stent thrombosis
Common triggers	Drugs, food	Drugs, contrast	Stent material
Treatment	Anti-allergic + vasodilators	Anti-allergic + PCI	Anti-allergic + stent management

## List of abbreviations

KS: Kounis syndrome
CAD: coronary artery disease
LAD: left anterior-descending artery
ICD: implantable cardioverter-defibrillator
BMI: body mass index
ECG: electrocardiogram
DCA: distal circumflex artery
RCA: right coronary artery
PCI: percutaneous coronary intervention
CCU: coronary care unit
ECHO: echocardiography
LVEF: left ventricular ejection fraction
LVDD: left ventricular diastolic diameter
ACS: acute coronary syndrome

## Author contribution

M.D., R.C., S.M.H., and J.N..: conceptualized the study, curated data, and conducted the investigation; M.D., R.C., M.E.K., S.M.H., A.K. and J.N.: wrote the original manuscript; A.K., N.Z.: reviewed and edited the manuscript; A.K., and J.N.: administered the project; J.N.: supervised the study.

## Patient consent

Written informed consent was obtained from the patient for publication of this case report and accompanying images.

## Ethical approval

Ethical approval was waived based on the observational nature of the report by our institution.

## Guarantor

Jamil Nasrallah.

## Research registration number

Not applicable.

## Funding

No funding was received.

## Conflict of interest statement

The authors declare no potential conflicts of interest with respect to the research, authorship, and/or publication of this article.
